# Clinical Factors Associated with Low-Contrast Visual Acuity after Reduced-Fluence Photodynamic Therapy in Patients with Resolved Central Serous Chorioretinopathy and Good Baseline Visual Acuity

**DOI:** 10.3390/ph14040303

**Published:** 2021-03-28

**Authors:** Aya Chubachi, Akiko Miki, Mayuka Hayashida, Mari Sakamoto, Hisanori Imai, Sentaro Kusuhara, Makoto Nakamura

**Affiliations:** Department of Surgery, Division of Ophthalmology, Kobe University Graduate School of Medicine, 7-5-2 Kusunoki-cho, Chuo-ku, Kobe 650-0017, Japan; achuba@med.kobe-u.ac.jp (A.C.); mh525@med.kobe-u.ac.jp (M.H.); mariwsakamoto@people.kobe-u.ac.jp (M.S.); hisimai@med.kobe-u.ac.jp (H.I.); kusu@med.kobe-u.ac.jp (S.K.); manakamu@med.kobe-u.ac.jp (M.N.)

**Keywords:** central serous chorioretinopathy, photodynamic therapy, contrast visual acuity

## Abstract

This retrospective study was conducted to investigate the clinical factors associated with low-contrast visual acuity after reduced-fluence photodynamic therapy (RFPDT) in patients with resolved central serous chorioretinopathy (CSC) and good baseline visual acuity. A total of 45 eyes of 45 patients with resolved CSC at post-RFPDT and best-corrected visual acuity of >1.0 (logarithm of the minimal angle of resolution [logMAR], 0) at baseline were examined. Visual acuities of both eyes were measured at four contrast levels (100%, 25%, 12%, and 6%) at post-RFPDT. The low-contrast visual acuity (6%, 12%, and 25%) was significantly lower than the 100% contrast visual acuity in the affected eyes. Visual acuities of affected eyes were significantly worse than those of fellow eyes at any contrast levels. The degree of changes in 6% and 100% contrast visual acuities was significantly greater in affected eyes than that in fellow eyes (*p* < 0.05). The 6% contrast visual acuities in affected eyes at post-RFPDT were significantly associated with the symptom duration (*p* < 0.05). Patients with a long duration of symptoms might have disturbed low-contrast visual acuities at post-RFPDT even if their baseline visual acuities were good.

## 1. Introduction

Central serous chorioretinopathy (CSC) is characterized by localized serous retinal detachment (SRD) at the macula [1,2], predominantly in middle-aged men. Patients with CSC often complain of visual symptoms, such as decreased contrast sensitivity, minor vision blurring, central scotoma, and metamorphopsia [1,2]. However, the underlying pathological mechanism of the disease remains unclear. In eyes with CSC, distinct choroidal abnormalities, such as thickened choroids, choroidal vascular hyperpermeability, filling delay, and venous congestion are visible on optical coherence tomography (OCT) and indocyanine green angiography (ICGA) [1,2]. In the pathogenesis of CSC, these abnormalities are believed to cause decompensation of the overlying retinal pigment epithelium, resulting in focal or diffuse breakdown of the outer retinal barrier as well as neuroretinal detachment [1,2].

In eyes with CSC, SRD is commonly resolved spontaneously within 3–4 months, whereas 30–50% of cases are chronic with persistent or recurrent SRD. Treatment is considered to prevent vision loss caused by damage to the foveal photoreceptors in eyes with chronic CSC [1,2]. Reduced-fluence photodynamic therapy (RFPDT) with modified standard photodynamic therapy (PDT) protocol has been reported to be effective for the complete resolution of SRD in eyes with CSC [3,4,5,6]. The most used modifications were as follows: (1) half-time PDT (PDT with standard-dose verteporfin (6 mg/mm^2^) and halved laser irradiation time (42 s)) and (2) half-dose PDT (PDT with a half-dose verteporfin (3 mg/mm^2^) and standard laser irradiation time (83 s)). In addition, RFPDT has also been reported to effectively improve not only visual acuities but also existing visual symptoms [7,8].

In clinical settings, visual acuities of eyes with active CSC are often preserved even after a reasonable follow-up period. It has been hypothesized that a diluted inter cone photoreceptor matrix that fills the detachment supports sufficient exchanges for a satisfactory visual function to be maintained [9]. Deciding the optimal treatment timing is sometimes difficult in patients with CSC and good visual acuities. By contrast, patients with resolved CSC frequently complain of visual symptoms such as decreased contrast sensitivities and metamorphopsia even with good visual acuities [10,11,12]. Hence, it is necessary to design a treatment intervention to avoid visual symptoms that disturb the quality of vision is necessary.

Low-contrast visual acuities were reportedly decreased in eyes with various ocular diseases, including ocular hypertension, glaucoma, and early diabetic retinopathy even when they had normal visual acuities [13]. To date, no studies have reported low-contrast visual acuities in eyes with CSC. Previous reports demonstrated that low-contrast visual acuity tests as well as contrast sensitivity function measurements can be useful [14]. Several studies have reported contrast sensitivity in eyes with CSC [10,12,15]. Contrast sensitivity has been reportedly decreased in both eyes with active CSC and eyes with resolved CSC as compared with fellow eyes [12,15]. Moreover, it has been reported that in eyes with resolved CSC, contrast sensitivity was decreased despite visual acuities being preserved [12,15,16]. However, no studies reported clinical factors associated with contrast sensitivity or low-contrast visual acuities in eyes with CSC. In this study, low-contrast visual acuities in patients with CSC were investigated at post-RFPDT and also investigated the association between low-contrast visual acuities and clinical factors at pre- and post-RFPDT.

## 2. Results

This study included a total of 45 eyes of 45 patients. Patients’ clinical characteristics are presented in Table 1. Their average age was 47.9 ± 8.3 years. The average symptom duration was 8.6 ± 12.5 months. All affected eyes had complete SRD resolution at post-RFPDT and visual acuities significantly improved at post-RFPDT (*p* < 0.002, Wilcoxon signed-rank test). All eyes did not require additional treatment during the follow-up period.

**Figure 1:**
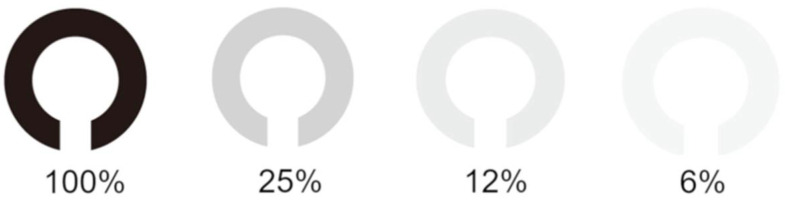
Contrast visual acuity charts. Depicted are low-contrast Landolt ring charts at 100%, 25%, 12%, and 6% concentrations.

In affected eyes, the contrast visual acuity was significantly decreased at 6%, 12%, and 25% contrast as compared to the normal-contrast visual acuity (100%), respectively (*p* < 0.001 at all low-contrast levels) (Figure 1, Table 2). In fellow eyes, the contrast visual acuity was also significantly decreased at 6%, 12%, and 25% contrast as compared to the normal-contrast visual acuity (100%), respectively (*p* < 0.001 at all low-contrast levels) (Table 2). Visual acuities of affected eyes were significantly worse than those of fellow eyes at any contrast levels (*p* = 0.01 at 100%; *p* < 0.01 at 25% and 6%; and *p* = 0.024 at 12% contrast) (Table 2).

No significant differences in the amount of changes in visual acuities were observed between 25% and 12% contrast and 100% contrast between affected and fellow eyes (Table 3). Conversely, the degree of changes in visual acuities between 6% and 100% contrast was significantly greater in affected eyes than that in fellow eyes (*p* < 0.05).

**Figure 2:**
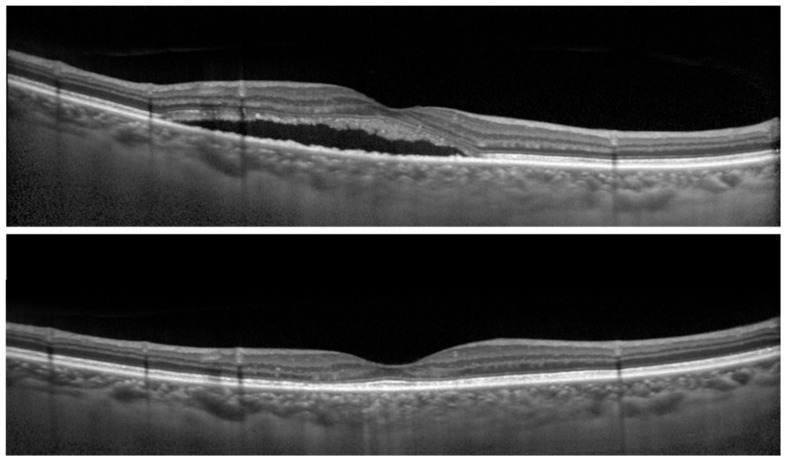
Optical coherence tomographic (OCT) images of the right eye of a 68-year-old man with central serous chorioretinopathy (CSC). (Top) Before reduced-fluence photodynamic therapy (RFPDT) and (Bottom) at 12 months after RFPDT. Complete resolution of SRD was observed after RFPDT. The 100% contrast visual acuity (decimal visual acuities) before and 12 months after RFPDT was 1.5, respectively. On the other hand, the 6% contrast visual acuity (decimal visual acuities) after RFPDT was 0.5. The symptom of duration was 11 months. The outer nuclear layer thicknesses before and after RFPDT were 69.0 and 92.0 µm, respectively.

Next, the association between 6% contrast visual acuity of affected eyes at post-RFPDT and clinical parameters including findings of OCT of affected eyes at pre- and post-RFPDT was investigated (Figure 2).

The 6% contrast visual acuities in affected eyes at post-RFPDT was significantly associated with symptom duration (*p* < 0.05) and the outer nuclear layer (ONL) thickness at pre-RFPDT showed a marginal significance (*p* = 0.052) (Table 4). Moreover, no significant association was observed between 6% contrast visual acuities at post-RFPDT and 100% contrast visual acuities at pre-RFPDT (Table 4).

## 3. Discussion

Patients with resolved CSC often complain of visual symptoms, such as decreased contrast sensitivity, minor vision blurring, central scotoma, and metamorphopsia despite of good visual acuities. Therefore, when treating patients with CSC, improving their visual acuity and symptoms, including contrast sensitivity, is essential. Till date, the low-visual acuities in eyes with CSC have not been investigated. Moreover, the relationship between the low-visual acuities and clinical parameters in eyes with CSC has not also been reported. In this study, we showed that low-contrast visual acuities were decreased in eyes with resolved CSC at post-RFPDT even if their visual acuities were good at baseline and found that low-contrast visual acuities at post-RFPDT were significantly correlated with the symptom duration. The low-contrast visual acuity test may be useful as another functional parameter of patients with CSC, particularly those with preserved visual acuities. To the best of our knowledge, this is the first study investigating the low-contrast visual acuities in eyes with CSC and the association between low-contrast visual acuities and clinical factors.

Plainis et al. [11] reported a patient with active CSC whose contrast sensitivity decreased even at maintained VA. Maaranen and Mäntyjärvi [16] investigated contrast sensitivity in patients who recovered from CSC with visual acuities of logMAR0 or better and found that contrast sensitivity was worse than the fellow eyes. Koskela et al. [12] also reported that contrast sensitivity decreased in eyes with previous history of CSC regardless of the normal visual acuities. Another study by Lourthai and Bhurayanontachai [15] showed that contrast sensitivity improved after the SRD resolution and did not regain its normal value despite of excellent final VA. Consistent with previous reports [12,16], low-contrast visual acuities decreased in affected eyes and were worse in affected eyes than fellow eyes after SRD resolution regardless of good baseline visual acuities in this study. Considering that visual acuities of affected eyes significantly improved at post-RFPDT, the low-contrast visual acuity test may be a more sensitive test than the normal visual acuity test.

In this study, 6% contrast visual acuities were significantly associated with symptom duration. This is a reasonable result because symptom duration approximately reflects the duration of SRD. Prasad and Divya [17] showed that patients with CSC with SRD for >3 months had disturbed vision due to loss of color and contrast sensitivity after the SRD resolution and proposed early intervention for patients with CSC. However, visual acuities of eyes with CSC are often preserved even after a reasonable follow-up period, and early intervention should be considered for the quality of vision even if visual acuities are normal. In patients with unknown symptom duration, especially in recurrent cases, the low-contrast visual acuity at pre RFPDT may help determine the timing of treatment.

A marginal significance was observed between 6% contrast visual acuities and ONL thickness in this study. Ozdemir et al. [18] investigated a relationship between the ONL thickness and symptom duration in patients with CSC and reported that the ONL thickness was negatively correlated with the symptom duration and positively correlated with the visual acuities. They concluded that photoreceptor loss could begin within the first 3 months of CSC. In the physiologic cycle, the retinal pigment epithelium (RPE) phagocytizes photoreceptor outer segment (OS) membranes that are shed as the renewal process of the OS. When the retina is detached, the OS is not phagocytized by RPE, resulting in photoreceptor cell apoptosis and thinning of ONL, which comprises cone cell bodies. The longer the SRD duration, the greater the damage to the photoreceptor cells, resulting in decreased low-contrast visual acuity.

In the univariate analysis, there was no significant difference between 6% contrast visual acuities at post-RFPDT and normal contrast visual acuities at pre-PDT. This might be because that we included only patients whose visual acuities were good at baseline in this study. A previous report [13] using low-contrast letter charts demonstrated that low-contrast charts detected visual loss in patients with early diabetic retinopathy showing abnormal fluorescein results even though visual acuity was normal. The low-contrast visual acuity test can detect a subclinical visual loss that does not affect the standard visual acuity test in eyes with good baseline visual acuities. The low-contrast visual acuity test may be useful as another functional parameter of patients with CSC, particularly those with preserved visual acuities.

Many researchers have shown that contrast sensitivity decreased in eyes with active or resolved CSC; meanwhile, no studies reported low-contrast visual acuity in eyes with CSC [10,11,12,15,16,17]. Fujita et al. [19] investigated the low luminal visual acuity in eyes with CSC and reported that low-luminance visual acuity was significantly worse in eyes with CSC than fellow eyes or age-matched normal eyes. Wood et al. [20] reported the difference between low-contrast and low-luminance visual acuities in eyes with choroideremia. Even in eyes with CSC, additional information could be provided by comparing low-contrast and low-luminance visual acuities in future studies.

In this study, low-contrast visual acuities at 6%, 12%, and 25% also reduced significantly compared with the normal-contrast visual acuities (100%) in fellow eyes, which is consistent with the results of previous reports [10,12,16]. Baran et al. [10] reported that contrast sensitivity in fellow eyes of patients with unilateral CSC was lower than that of normal eyes and found that the 19.4% of fellow eyes had RPE changes. In this study, there were no eyes showing RPE changes in fellow eyes. CSC is a bilateral disease that shares a similar clinical phenotype in both eyes, including the thick choroid [21,22]. The fellow eyes have a risk of CSC, and subclinical changes could remain after a previous CSC attack without significant visual disturbance in fellow eyes.

All affected eyes in this study were treated with RFPDT. In the univariate analysis, low-contrast visual acuities at post-PDT did not correlate with PDT spot size in this study. Several complications after conventional PDT, including RPE atrophy and choriocapillaris ischemia have been reported [23]. RFPDT can minimize these adverse effects. Karakus et al. [24] investigated contrast sensitivity changes before and after half-dose PDT in eyes with chronic CSC and demonstrated that treatment with half-dose PDT improved contrast sensitivity in eyes with chronic CSC. Therefore, the effect of PDT does not need to be considered for the difference between affected eyes and fellow eyes in low-contrast visual acuities.

This study has several limitations, including the small number of patients and short-term follow-up. Another limitation is that we used fellow eyes as control. Future studies with a large sample size and long-term follow-up are needed.

## 4. Materials and Methods

The study protocol adhered to the tenets of the Declaration of Helsinki and was approved by the institutional review board of Kobe University Hospital. Informed consent was obtained by using the opt-out method on the center’s website.

A total of 45 eyes of 45 consecutive patients with resolved CSC at post-RFPDT who were treated between December 2015 and July 2019 at Kobe University Hospital were retrospectively reviewed. The inclusion criteria were as follows: (1) The best-corrected visual acuity of ≥1.0 (logarithm of the minimal angle of resolution [logMAR], 0) at baseline; (2) the presence of SRD involving the fovea in OCT images at baseline, (3) the presence of angiographic leakage caused by CSC detected on fluorescein angiography (FA) at baseline, and (4) the presence of abnormally dilated choroidal vessels detected on ICGA at baseline. The exclusion criteria were: (1) Severe myopic or hyperopic eyes with refractive errors (spherical equivalent) of more than six diopters; (2) evidence of choroidal neovascularization; (3) presence of any other ocular disease that could affect visual acuity, including tilted disc syndrome, dome-shaped macula, and uveitis; (4) history of laser photocoagulation, transpupillary thermotherapy, or anti-vascular endothelial growth factor treatment; (5) presence of media opacities, such as cataracts, which could interfere in acquiring high-quality OCT, FA, and ICGA images; (6) presence of any other ocular disease that could affect contrast visual acuity including glaucoma and other retinal diseases including diabetic retinopathy; and (7) previous history of any ocular diseases in fellow eyes.

Clinical data with regard to age, sex, and symptom duration were collected. At baseline, all patients underwent a complete ophthalmologic examination, including slit-lamp examination, dilated fundus examinations and standard visual acuity measurements. Visual acuities of both eyes were measured at four contrast levels (100%, 25%, 12%, and 6%) of Landolt ring chart (Figure 1) after RFPDT (average 12.04 ± 12.15 M) using VC-60 visual acuity test (Takaji Seiko, Nagano, Japan). SRD was resolved in all eyes at the time of contrast visual acuity measurements.

**Figure 3:**
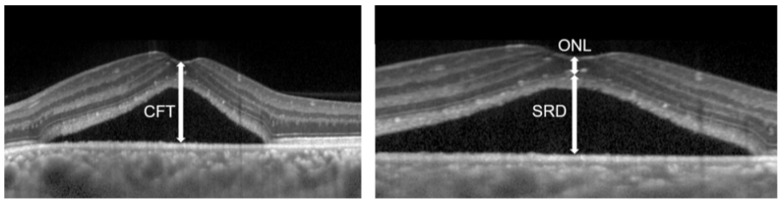
Optical coherence tomographic image of eyes with central serous chorioretinopathy.

The Spectralis OCT system (Heidelberg Spectralis OCT; Heidelberg Engineering GmbH, Heidelberg, Germany) was used to obtain macular scans. The Spectralis OCT system (Heidelberg Spectralis HRA2; Heidelberg Engineering GmbH) was used for FA and ICGA. Clinical data at pre- and post-treatment were recorded. For Spectralis OCT examinations, average values were obtained for horizontal and vertical line scans through the fovea. At baseline, the central foveal thickness (CFT) was measured from the inner surface of the neurosensory retina to the outer surface of RPE at the fovea (Figure 3). The SRD height was defined as the distance between the outer surface of the sensory retina and inner surface of RPE at the fovea (Figure 3). ONL thickness was measured as the distance between the outer surface of the inner limiting membrane and inner surface of the external limiting membrane (ELM) at the central fovea (Figure 3). Disruption of the ELM layer and the ellipsoid zone (EZ) layer within 500 μm from the fovea was also evaluated. Eyes in which these findings were detected in at least one scan of horizontal and vertical line scans were classified as having the presence of these findings. Since evaluating the EZ layer disruption at baseline was sometimes difficult because of the SRD or elongated photoreceptor OS, we evaluated it only at post-RFPDT.

Horizontal optical coherence tomography images of eyes with central serous chorioretinopathy, the serous retinal detachment (SRD) height, the central foveal thickness (CFT), and the outer nuclear layer (ONL) thickness are shown.

All patients were infused with verteporfin (Visudyne; Novartis, Basel, Switzerland) at 6 mg/m^2^ body surface area over 10 min; laser treatment was administered 15 min after the infusion. The standard light intensity was 600 mW/cm^2^, and the irradiation time was shortened to 42 s (half-time PDT). The spot size covered areas with active leaking spots on FA images.

The decimal visual acuity was converted into logMAR units for statistical analyses. Differences in contrast visual acuities among various concentrations were analyzed using the Wilcoxon signed-rank test. Differences in contrast visual acuities between affected and fellow eyes were analyzed using the Mann–Whitney U test. The amount of changes in contrast visual acuities between affected and fellow eyes was analyzed using the Mann–Whitney U test. Spearman’s rank correlation test was used to analyze the association between 6% contrast visual acuities after RFPDT and clinical parameters at pre or post RFPDT in affected eyes. A *p*-value of <0.05 was considered statistically significant. Statistical analyses were performed using the SPSS software, version 24.0 (IBM Corp., Armonk, NY, USA).

## 5. Conclusions

Patients with CSC with long symptom duration might have disturbed low-contrast visual acuities at post-RFPDT even if their baseline visual acuities are good. The low-contrast visual acuity test may be useful as another functional parameter of patients with CSC, particularly those with preserved visual acuities.

## Figures and Tables

**Table 1:** Clinical characteristics.

Clinical Charecteristics	Results
Case number, *n*	45
Age, years	47.9 ± 8.3
Sex (male/female)	32/13
Duration of symptoms, months	8.6 ± 12.5
Baseline VA, logMAR	−0.08 ± 0.10
Spot size, μm	3978.0 ± 863.1
ONL thickness, μm	95.2 ± 19.9
CFT, μm	369.0 ± 130.5
Height of SRD, μm	173.8 ± 128.8
Disruption of ELM layer (+/−)	3/42

VA, visual acuity; logMAR, logarithm of the minimum angle of resolution; ONL, outer nuclear layer; CFT, central foveal thickness; SRD, serous retinal detachment; ELM, external limiting membrane.

**Table 2:** Contrast visual acuities in affected and fellow eyes after reduced-fluence photodynamic therapy.

Index Concentration, %	VA	*p*-Value * vs. 100%	VA	*p*-Value ** vs. 100%	*p*-Value *** Affected vs. Fellow
	Affected Eye		Fellow Eye		
100	−0.13 ± 0.08		−0.18 ± 0.08		0.01
25	−0.01 ± 0.11	<0.001	−0.08 ± 0.09	<0.001	<0.01
12	0.10 ± 0.19	<0.001	0.03 ± 0.09	<0.001	0.024
6	0.15 ± 0.14	<0.001	0.18 ± 0.07	<0.001	<0.01

VA, visual acuity; logMAR, logarithm of the minimum angle of resolution. *, ** Wilcoxon signed-rank test. *** Mann–Whitney U test.

**Table 3:** Amount of changes in contrast visual acuities between affected and fellow eyes.

Index Concentration	Affected Eye	Fellow Eye	*p*-Value *
100–25%	0.12 ± 0.071	0.098 ± 0.062	0.279
100–12%	0.24 ± 0.13	0.21 ± 0.069	0.420
100–6%	0.37 ± 0.13	0.32 ± 0.11	0.038

* Mann–Whitney U test.

**Table 4:** Correlation between 6% contrast visual acuities after reduced-fluence photodynamic therapy and clinical parameters at pre- and post-treatments.

Clinical Parameters	r	*p*-Value *
Age, years	0.147	0.334
Symptom duration, months	0.299	0.048
PDT spot size, μm	0.148	0.331
VA before PDT, logMAR	−0.068	0.656
ONL thickness before PDT, μm	−0.292	0.052
ONL thickness after PDT, μm	−0.207	0.173
CFT before PDT, μm	−0.097	0.520
CFT after PDT, μm	−0.132	0.389
Height of SRD before PDT, μm^2^	0.003	0.985
Disruption of ELM layer, before PDT (+/−)	−0.035	0.821
Disruption of ELM layer, after PDT (+/−)	0.131	0.390
Disruption of EZ layer, after PDT (+/−)	0.209	0.168

PDT, photodynamic therapy; VA, visual acuity; logMAR, logarithm of the minimum angle of resolution; ONL, outer nuclear layer; CFT, central foveal thickness; SRD, serous retinal detachment; ELM, external limiting membrane; EZ, ellipsoid zone. * Spearman’s rank correlation coefficient.

## Data Availability

The data presented in this study are available on request from the corresponding author. The data are not publicly available due to privacy.
